# Understanding the psychiatric symptoms of COVID-19: a meta-analysis of studies assessing psychiatric symptoms in Chinese patients with and survivors of COVID-19 and SARS by using the Symptom Checklist-90-Revised

**DOI:** 10.1038/s41398-021-01416-5

**Published:** 2021-05-17

**Authors:** Qin Xie, Xiao-Bo Liu, Yan-Min Xu, Bao-Liang Zhong

**Affiliations:** 1 Department of Psychiatry, Wuhan Mental Health Center, Wuhan, Hubei province China; 2 Department of Psychiatry, Affiliated Wuhan Mental Health Center, Tongji Medical College of Huazhong University of Science & Technology, Wuhan, Hubei Province China

**Keywords:** Depression, Human behaviour

## Abstract

Understanding the psychiatric symptoms of COVID-19 could facilitate the clinical management of COVID-19 patients. However, the profile of psychiatric symptoms among COVID-19 patients has been understudied. We performed a meta-analysis of studies assessing psychiatric symptoms of COVID-19 and SARS patients and survivors by using the Symptom Checklist-90-Revised (SCL-90-R), an instrument covering a wide spectrum of psychiatric symptoms. Studies reporting SCL-90-R subscale scores among patients with and survivors of COVID-19 and SARS were retrieved from major English and Chinese literature databases. Patients’ pooled SCL-90-R subscale scores were compared to the Chinese normative SCL-90-R data, and Cohen’s *d* values were calculated to indicate the severity of psychiatric symptoms. The Joanna Briggs Institute Critical Appraisal Checklist for Studies Reporting Prevalence Data was used to assess the quality of the included studies. The search yielded 25 Chinese studies with 1675 acute COVID-19 and 964 acute SARS patients, 30 COVID-19 and 552 SARS survivors during very early recovery (up to 1 month since discharge), 291 SARS survivors during early recovery (1–6 months after discharge), and 48 SARS survivors during late recovery (12 months after discharge). None of the included studies were rated as good quality. The ten SCL-90-R-defined psychiatric symptoms, which were of medium-to-severe severity (*d* = 0.68–3.01), were all exhibited in acute COVID-19 patients, and the severity of these symptoms decreased to mild-to-medium during very early recovery (*d* = 0.17–0.73). SARS patients presented eight psychiatric symptoms with mild-to-severe severity during the acute stage (*d* =0.43–1.88), and thereafter, the severity of symptoms decreased over the follow-up period. However, somatization (*d* = 0.30) and anxiety (*d* = 0.28) remained at mild levels during late recovery. A wide variety of severe psychiatric symptoms have been reported by acute COVID-19 patients, and these symptoms, despite decreasing in severity, persist in very early recovery. The changing trajectory observed with SARS suggests that psychiatric symptoms of COVID-19 may persist for a long time after discharge, and therefore, periodic monitoring of psychiatric symptoms, psychosocial support, and psychiatric treatment (when necessary) may be necessary for COVID-19 patients from the acute to convalescent stages.

## Introduction

Psychiatric presentations and mental disorders are common among COVID-19 patients^1,2^. Empirical data have shown that 43.1% and 40.2% of COVID-19 patients suffer from depressive symptoms and mental illnesses, respectively^3,4^. Cooccurring mental health problems complicate the respiratory management of COVID-19 patients and negatively affect the prognosis of COVID-19 (refs. ^5,6^). For example, patients who develop psychotic symptoms may not adhere to respiratory treatment or may even threaten the safety of frontline medical staff, and depressed survivors may not be able to return to work. Therefore, a timely and detailed assessment of mental health morbidities is important for the effective clinical management of COVID-19 patients and survivors.

Available research on mental health problems associated with COVID-19 is limited to case reports/series, self-report questionnaire surveys, and mental disorder surveys^3,4,7–10^. Because case reports/series are subject to selection bias, i.e., reporting unusual cases with manic episodes, the generalizability of their findings is poor. Most prior questionnaire surveys focused on depressive and anxiety symptoms, so data regarding psychiatric symptoms other than depression and anxiety associated with COVID-19 are still limited. Despite having first-hand data on a variety of mental disorders, mental disorder surveys have provided little information on subclinical psychiatric symptoms of COVID-19. According to the mental health continuum model, psychiatric symptoms are early signs of mental disorders, and persons with long-lasting and severe symptoms are more likely to develop mental disorders^11^. Therefore, the assessment and identification of psychiatric symptoms have clinical implications for early psychological interventions for COVID-19 patients. To the best of our knowledge, only one study has assessed psychiatric symptoms of COVID-19 patients by using psychiatric interviews, and 11 symptoms were identified in this patient population, including insomnia, aggressive behaviors, delusions, and hallucinations^5^. However, because the sample size of this study was small (*n* = 25) and its participants were COVID-19 patients who received psychiatric inpatient care for comorbid first-onset mental disorders, its findings are difficult to generalize to general COVID-19 patients. Therefore, data regarding the full spectrum of psychiatric symptoms among persons with COVID-19 are still very limited.

As of March 25, 2021, the total number of globally confirmed cases of COVID-19 had been 125,436,393, of whom 10,130,4931 survived, making postdischarge rehabilitation services an urgent clinical priority^12–14^. Unfortunately, the long-term mental health sequelae of COVID-19 are still poorly understood due to the lack of empirical studies^15,16^.

The Symptom Checklist-90-Revised (SCL-90-R) is a widely used self-report scale to assess a broad range of psychological problems and symptoms of psychopathology^17^. The SCL-90-R has been used to assess the psychiatric symptoms of both clinical and nonclinical populations, including SARS patients^18^. There have been some studies investigating psychiatric symptoms of COVID-19 by using the SCL-90-R^19^. This provided us with an opportunity to examine the profile of psychiatric symptoms of COVID-19; however, these studies were limited by their small sample sizes and wide variations in SCL-90-R test results^20–22^. For example, Xing and colleagues assessed the psychiatric symptoms of 42 acute COVID-19 patients and reported that the mean score of the depression subscale of the SCL-90-R was 1.36, while Wang and colleagues investigated 40 acute COVID-19 patients and found the corresponding mean score was 3.15, which was an over twofold difference^21,23^.

To fill the abovementioned knowledge gap, we performed a meta-analysis of clinical studies assessing psychiatric symptoms in COVID-19 patients, as denoted by the SCL-90-R scores. Given that COVID-19 and SARS have similar pathophysiological mechanisms, we also quantitatively combined the SCL-90-R test results of SARS patients, in particular SARS survivors, which may inform the planning of follow-up services for COVID-19 survivors.

## Methods

### Inclusion and exclusion criteria

The inclusion criteria were as follows: (a) studies were cross-sectional studies or baseline assessments of cohort or interventional studies published in English or Chinese; (b) study participants were adults with a current or past diagnosis of COVID-19 or SARS; (c) studies included a minimum sample of ten patients; (d) participants’ psychiatric symptoms were assessed with the SCL-90-R; and (e) studies presented the mean scores [with standard deviations (SDs)] of at least one of the ten primary subscales of the SCL-90-R: somatization, obsessive-compulsive, interpersonal sensitivity, depression, anxiety, hostility, phobic anxiety, paranoid ideation, psychoticism, and appetite and sleep. We excluded duplicate reports and studies using samples of COVID-19 patients referred for psychiatric consultation.

### Literature search

Major Chinese and English databases were searched from their inception to March 1, 2021. These databases were China National Knowledge Infrastructure, Wanfang Data Knowledge Service Platform, CQVIP, SinoMed, PubMed, EMBASE, and PsycInfo. The following search terms were used: “COVID-19” or “2019-nCoV” or “coronavirus” or “SARS” or “severe acute respiratory syndrome” and “SCL-90” or “Symptom Checklist”. Reference lists of the retrieved papers were also hand-searched to avoid missing certain records.

### Data extraction

Variables collected from each included study were first author, publication year, coronavirus disease (COVID-19 vs. SARS), type of participants (patients vs. survivors), sample size, numbers of male and female participants, and SCL-90-R subscale scores (mean ± SD). In accordance with Gardner and Moallef^24^, the stage of coronavirus disease was roughly divided into the acute stage (i.e., at admission and during hospital stay), very early recovery stage (at discharge and up to 1 month after discharge), early recovery stage (1–6 months postdischarge), and late recovery stage (6–12 months postdischarge).

### Quality assessment

The Joanna Briggs Institute Critical Appraisal Checklist for Studies Reporting Prevalence Data (abbreviated as “JBI checklist”) was used to assess the quality of the included studies^25^. The JBI checklist has nine methodological items, with each having four answer choices (yes, no, unclear, or not applicable): sample frame, sampling, sample size, description of subjects and setting, sample coverage of the data analysis, validity of the method for assessing the outcome, standardization and reliability of the method for assessing outcome, statistical analysis, and response rate. The quality score is the total number of “yes” answers, which can range from 0 to 9, with higher scores suggesting higher quality^26^. A JBI score of “9” suggests “good quality”.

The first and second authors independently performed the literature search, selected eligible studies, extracted data, and assessed the quality of the included studies. Any discrepancies were resolved by their consensus.

### Statistical analysis

We used the “metamean” package of R 4.0.2 to produce pooled estimates and their 95% confidence intervals (CIs) for the SCL-90-R subscale scores, according to coronavirus disease (COVID-19 vs. SARS) and stage of coronavirus disease (acute vs. recovery). When there was little evidence of heterogeneity (*I*^2^ ≤ 50%), a fixed effects model was used to produce the combined estimates; otherwise, a random effects model was used. Publication bias was tested by using Egger’s test when the total number of included studies was ten or more for the meta-analysis of a SCL-90-R subscale; otherwise, publication bias was not tested. Due to the small number of included studies, sources of heterogeneity in the meta-analyses were not explored. To gauge magnitudes of differences in symptom severity between patients and the general population, Cohen’s *d* values were calculated for SCL-90-R subscale scores, with 0.20–0.49, 0.50–0.79, and 0.80+ being operationally defined as mild, medium, and severe symptoms, respectively^27^. We introduced two Chinese SCL-90-R normative data in 2006 and 2014, as the comparison references for the SCL-90-R scores of SARS patients and survivors, and COVID-19 patients and survivors, respectively^28,29^.

## Results

**Fig. 1:**
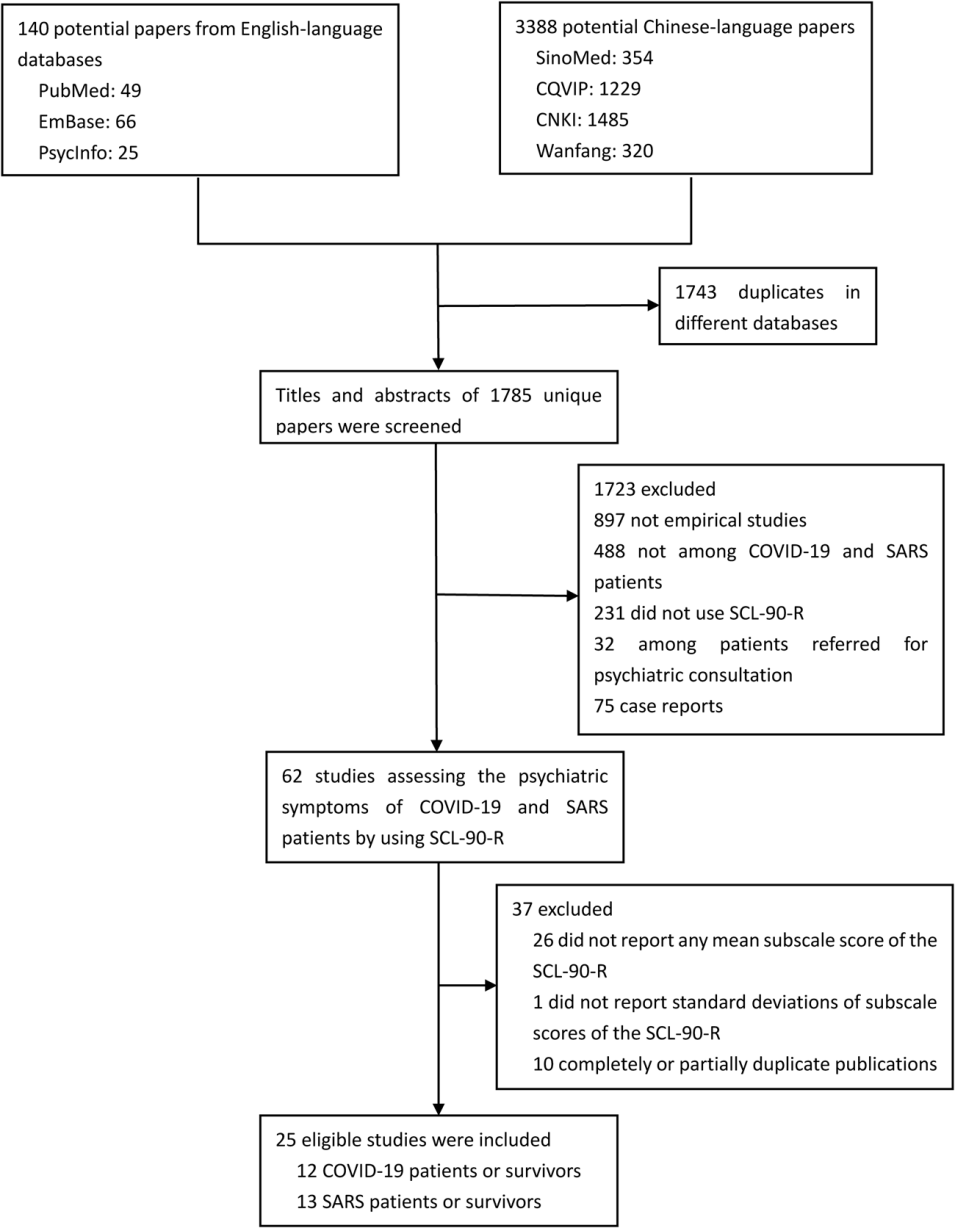
Study identification and inclusion. Flowchart depicting the process of searching and screening for eligible studies of this meta-analysis.

The process of study identification and inclusion is shown in Fig. 1. Finally, 25 studies were eligible for this study, including 12 studies with 1675 acute patients with COVID-19, 2 studies with 30 COVID-19 survivors during very early recovery, 7 studies with 964 acute patients with SARS, 7 studies with 552 SARS survivors during very early recovery, 4 studies with 291 SARS survivors during early recovery, and 1 study with 48 SARS survivors during late recovery^20–23,30–50^. Participants in the included studies were all Chinese patients or survivors. The quality scores of the included studies ranged from 2 to 7. Detailed characteristics and quality assessment of the included studies are displayed in Table 1.

**Table 1:** Characteristics and quality of included studies.

Study	Study site	Sample size	% of females	Age (years)	Survey method	Sampling method	Infected medical staff	% of severe and critical patients	Assessment time-point	The Joanna Briggs Institute Critical Appraisal Checklist for Studies Reporting Prevalence Data									
										Q1	Q2	Q3	Q4	Q5	Q6	Q7	Q8	Q9	Quality score
*COVID-19 patients during the acute stage*																			
Hu et al.^30^	A shelter hospital in Wuhan, China	356	47.2	Mean: 44.4	O	C	N	0.0	Hospitalization	U	N	Y	N	Y	Y	U	Y	Y	5
Hui et al.^20^	A designated hospital in Nanyang, China	72	45.8	Mean: 57.6	O	C	N	6.9	Hospitalization	Y	N	N	Y	Y	Y	U	Y	U	5
Liu and Yu^31^	A designated hospital in Wuhan, China	97	NR	Mean: 57.6	PP	C	N	No critical	Hospitalization	Y	N	N	N	Y	Y	U	Y	Y	5
Mei et al.^32^	A designated hospital in Wuhan, China	70	78.6	Mean: 36.2	O	C	Y	11.4	Hospitalization	N	N	N	Y	Y	Y	U	Y	U	4
Qian et al.^22^	A designated hospital in Kunming, China	16	43.8	NR	O	C	N	22.2	Hospitalization	Y	N	N	N	Y	Y	U	Y	Y	5
Qin et al.^33^	A designated hospital in Changsha, China	112	56.3	Median: 40	O	C	N	10.7	Hospitalization	N	N	Y	Y	Y	Y	U	Y	U	5
Wang et al.^34^	A designated hospital in Wuhan, China	652	46.9	Mean: 51.5	O	C	N	No critical	Hospitalization	N	N	Y	Y	Y	Y	U	Y	Y	6
Wang et al.^23^	A designated hospital in Xi’an, China	40	45.0	Mean: 46.0	O	C	N	100.0	Hospitalization	N	N	N	Y	U	Y	U	Y	U	3
Xi^35^	A designated hospital in Wuhan, China	20	60.0	Mean: 45.0	O	C	N	NR	Hospitalization	N	N	N	Y	U	Y	U	Y	U	3
Xing et al.^21^	A designated hospital in Ningbo, China	42	64.3	Mean: 46.6	PP	C	N	31.0	Hospitalization	U	N	N	Y	Y	Y	U	Y	Y	5
Yang et al.^36^	A shelter hospital in Wuhan, China	198	56.6	Mean: 37.7	O + PP	C	N	0.0	Hospitalization	N	N	Y	Y	U	Y	Y	Y	U	5
*COVID-19 survivors during the very early recovery stage*																			
Gong et al.^37^	Wuhan, China	14	85.7	Mean: 33.0	O	C	Y	NA	15 days after discharge	N	N	N	N	U	Y	U	Y	U	2
Qian et al.^22^	Kunming, China	16	43.8	NR	O	C	N	NA	A week after discharge	Y	N	N	N	Y	Y	U	Y	Y	5
*SARS patients during the acute stage*																			
Peng et al.^38^	A designated hospital in Taiyuan, China	117	53.8	Mean: 36.5	PP	C	N	NR	Admission	N	N	Y	Y	Y	Y	U	Y	Y	6
Sun and Ren^39^	A designated hospital in Beijing, China	35	88.6	Mean: 30.2	PP	C	Y	NR	Hospitalization	N	N	N	Y	Y	Y	U	Y	U	4
Wang et al.^40^	A designated hospital in Beijing, China	669	45.0	Mean: 35.0	PP	CL	N	6.9	Admission	Y	Y	Y	Y	Y	Y	U	Y	U	7
Wang et al.^41^	A designated hospital in Beijing, China	40	NR	NR	PP	R	N	NR	Hospitalization	U	Y	N	N	Y	Y	U	Y	U	4
Wang et al.^42^	A designated hospital in Beijing, China	29	48.3	Mean: 37.5	PP	C	N	NR	Admission	N	N	N	Y	Y	Y	U	Y	U	4
Xiao et al.^43^	A designated hospital in Beijing, China	31	45.2	Mean: 32.0	PP	C	N	NR	Hospitalization	Y	N	N	Y	Y	Y	U	Y	U	5
Yang^44^	A designated hospital in Taiyuan, China	43	41.9	Mean: 34.5	PP	C	N	NR	Admission	Y	N	N	N	Y	Y	U	Y	N	4
*SARS survivors during the very early stage of recovery*																			
Hu et al.^45^	Beijing, China	35	42.9	Mean: 32.0	PP	C	N	NA	At discharge	Y	N	N	Y	Y	Y	U	Y	U	5
Peng et al.^38^	Taiyuan, China	117	53.8	Mean: 36.5	PP	C	N	NA	At discharge	N	N	Y	Y	Y	Y	U	Y	Y	6
Liu et al.^46^	Tianjing, China	48	70.8	NR	PP	C	N	NA	At discharge	N	N	N	U	Y	Y	U	Y	N	3
Wang et al.^40^	Beijing, China	177	NR	NR	PP	C	N	NA	At discharge	Y	N	Y	Y	Y	Y	U	Y	U	6
Wang et al.^47^	Beijing, China	103	59.2	≤40:79.6%	PP	C	N	NA	At discharge	Y	N	Y	Y	Y	Y	U	Y	Y	7
Wang et al.^42^	Beijing, China	29	48.3	Mean: 37.5	PP	C	N	NA	At discharge	N	N	N	Y	Y	Y	U	Y	U	4
Yang^44^	Taiyuan, China	43	41.9	Mean: 34.5	PP	C	N	NA	At discharge	Y	N	N	N	Y	Y	U	Y	N	4
*SARS survivors during the early stage of recovery*																			
Gao et al.^48^	Tianjin, China	67	68.7	Mean: 25.3	PP	C	N	NA	6 months after discharge	Y	N	N	N	U	Y	U	Y	N	3
Kuang et al.^49^	Guangzhou, China	62	54.8	Median: 37	PP	C	N	NA	6 months after discharge	N	N	N	U	Y	Y	U	Y	U	3
Lin et al.^50^	Guangzhou, China	45	55.6	Mean: 35.0	PP	C	N	NA	3–6 months after discharge	N	N	N	Y	Y	Y	U	Y	N	4
Peng et al.^38^	Taiyuan, China	117	53.8	Mean: 36.5	PP	C	N	NA	3 months after discharge	N	N	Y	Y	Y	Y	U	Y	Y	6
*SARS survivors during the late stage of recovery*																			
Liu et al.^46^	Tianjing, China	48	70.8	NR	PP	C	N	NA	12 months after discharge	N	N	N	U	Y	Y	U	Y	N	3

Note: Q1: Was the sample representative of the target population?; Q2: Were study participants recruited in an appropriate way?; Q3: Was the sample size adequate? (The minimum sample size was 100 for the current quality assessment); Q4: Were the study subjects and setting described in detail?; Q5: Is the data analysis conducted with sufficient coverage of the identified sample?; Q6: Were objective, standard criteria used for measurement of the condition?; Q7: Was the condition measured reliably?; Q8: Was there appropriate statistical analysis?; Q9: Was the response rate adequate, and if not, was the low response rate managed appropriately?

*NR* not reported, *PP* paper–pencil self-administered questionnaire, *O* online self-administered questionnaire, *C* convenience sampling, *CL* cluster sampling, *R* random sampling, *NA* not applicable, *N* not, *Y* Yes, *U* unclear.

**Fig. 2:**
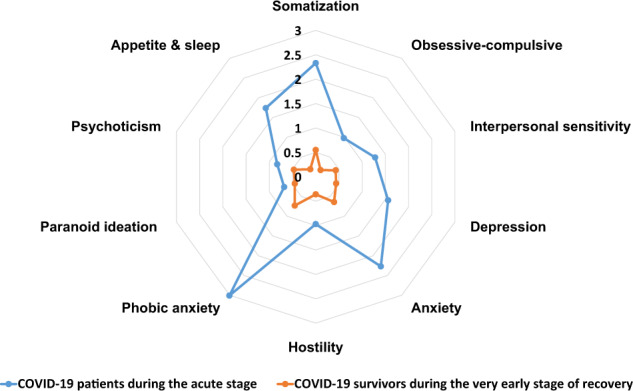
Severity of psychiatric symptoms of COVID-19 patients and survivors. Radar map depicting the severity of psychiatric symptoms of COVID-19 patients and survivors, as measured by Cohen’s d values.

Acute COVID-19 patients presented all the ten SCL-90-R-defined psychiatric symptoms. Nearly all the psychiatric symptoms of COVID-19 were severe during the acute stage, and their severity decreased to mild-to-medium during very early recovery: somatization (*d* = 2.33 and 0.55, respectively), obsessive-compulsive (*d* = 0.98 and 0.17), interpersonal sensitivity (*d* = 1.28 and 0.43), depression (*d* = 1.56 and 0.44), anxiety (*d* = 2.27 and 0.64), hostility (*d* = 0.97 and 0.36), phobia (*d* = 3.01 and 0.73), paranoid ideation (*d* = 0.68 and 0.45), psychoticism (*d* = 0.83 and 0.47), and appetite and sleep (*d* = 1.74 and 0.19; Table 2 and Fig. 2).

**Table 2:** Results of meta-analysis of subscale scores of the Symptom Checklist-90-Revised (SCL-90-R) and the severity of psychiatric symptoms, as indicated by Cohen’s *d* values.

Psychiatric symptoms	Number of studies	Sample size	Heterogeneity, I2 (%), *P*	Patient sample		Chinese SCL-90-R norms		Cohen’s *d*	*Z*	*P*	Egger’s test, *t*, *P*
				Pooled mean (95% CI)	Standard deviation	Mean	Standard deviation				
*COVID-19 patients during the acute stage*											
Somatization	11	1675	99.9, <0.001	2.17 (1.86, 2.48)	0.16	1.37	0.46	2.33	141.39	<0.001	0.187, 0.856
Obsessive-compulsive	9	1003	99.6, <0.001	2.07 (1.81, 2.33)	0.13	1.66	0.58	0.98	61.33	<0.001	NA
Interpersonal sensitivity	9	1003	99.6, <0.001	2.03 (1.71, 2.35)	0.16	1.51	0.55	1.28	72.09	<0.001	NA
Depression	10	1655	99.9, <0.001	2.08 (1.65, 2.52)	0.22	1.45	0.53	1.56	87.18	<0.001	0.360, 0.728
Anxiety	11	1675	99.7, <0.001	2.23 (1.86, 2.59)	0.19	1.4	0.48	2.27	130.90	<0.001	1.008, 0.340
Hostility	10	1023	99.7, <0.001	1.89 (1.53, 2.25)	0.19	1.48	0.57	0.97	52.87	<0.001	1.369, 0.208
Phobic anxiety	8	933	99.7, <0.001	2.10 (1.86, 2.33)	0.12	1.23	0.39	3.01	163.57	<0.001	NA
Paranoid ideation	8	933	99.9, <0.001	1.69 (1.10, 2.27)	0.30	1.41	0.50	0.68	25.82	<0.001	NA
Psychoticism	8	933	99.8, <0.001	1.68 (0.94, 2.42)	0.38	1.34	0.44	0.83	26.13	<0.001	NA
Appetite and sleep	4	800	97.1, <0.001	2.27 (1.83, 2.72)	0.23	1.51	0.58	1.74	80.12	<0.001	NA
*COVID-19 survivors during the very early stage of recovery*											
Somatization	2	30	94.7, <0.001	1.59 (0.96, 2.21)	0.32	1.37	0.46	0.55	3.70	<0.001	NA
Obsessive-compulsive	2	30	98.0, <0.001	1.75 (0.84, 2.66)	0.47	1.66	0.58	0.17	1.03	0.234	NA
Interpersonal sensitivity	2	30	97.0, <0.001	1.73 (0.82, 2.64)	0.47	1.51	0.55	0.43	2.55	0.016	NA
Depression	2	30	97.2, <0.001	1.66 (0.84, 2.48)	0.42	1.45	0.53	0.44	2.72	0.010	NA
Anxiety	2	30	94.8, <0.001	1.67 (0.98, 2.35)	0.35	1.4	0.48	0.64	4.18	<0.001	NA
Hostility	2	30	95.6, <0.001	1.65 (0.96, 2.33)	0.35	1.48	0.57	0.36	2.64	0.012	NA
Phobic anxiety	2	30	96.2, <0.001	1.49 (0.86, 2.12)	0.32	1.23	0.39	0.73	4.47	<0.001	NA
Paranoid ideation	2	30	96.3, <0.001	1.60 (0.95, 2.25)	0.33	1.41	0.5	0.45	3.17	0.003	NA
Psychoticism	2	30	99.0, <0.001	1.53 (0.82, 2.23)	0.36	1.34	0.44	0.47	2.85	0.007	NA
Appetite and sleep	1	16	NA	1.43 (1.32, 1.54)	0.06	1.51	0.58	0.19	5.43	<0.001	NA
*SARS patients during the acute stage*											
Somatization	7	964	95.5, <0.001	1.96 (1.69, 2.23)	0.14	1.42	0.44	1.65	48.71	<0.001	NA
Obsessive-compulsive	7	964	98.3, <0.001	1.83 (1.43, 2.23)	0.21	1.66	0.52	0.43	12.44	<0.001	NA
Interpersonal sensitivity	7	964	99.1, <0.001	1.72 (1.34, 2.10)	0.19	1.51	0.49	0.55	15.98	<0.001	NA
Depression	7	964	98.1, <0.001	2.01 (1.69, 2.33)	0.16	1.50	0.47	1.44	42.12	<0.001	NA
Anxiety	7	964	99.3, <0.001	2.01 (1.38, 2.64)	0.32	1.34	0.39	1.88	49.14	<0.001	NA
Hostility	7	964	97.8, <0.001	1.65 (1.38, 1.92)	0.14	1.49	0.51	0.43	12.79	<0.001	NA
Phobic anxiety	7	964	99.7, <0.001	1.68 (1.05, 2.31)	0.32	1.27	0.39	1.15	30.03	<0.001	NA
Paranoid ideation	7	964	98.6, <0.001	1.45 (1.22, 1.69)	0.12	1.44	0.47	0.04	1.27	0.178	NA
Psychoticism	7	964	98.9, <0.001	1.50 (1.17, 1.83)	0.17	1.33	0.39	0.56	16.08	<0.001	NA
*SARS survivors during the very early stage of recovery*											
Somatization	7	552	83.8, <0.001	1.74 (1.58, 1.90)	0.08	1.42	0.44	1.01	30.02	<0.001	NA
Obsessive-compulsive	7	552	93.7, <0.001	1.79 (1.55, 2.02)	0.12	1.66	0.52	0.33	9.67	<0.001	NA
Interpersonal sensitivity	7	552	95.4, <0.001	1.66 (1.40, 1.92)	0.13	1.51	0.49	0.41	11.57	<0.001	NA
Depression	7	552	95.6, <0.001	1.76 (1.47, 2.05)	0.15	1.50	0.47	0.74	20.52	<0.001	NA
Anxiety	7	552	89.3, <0.001	1.70 (1.51, 1.89)	0.10	1.34	0.39	1.27	36.63	<0.001	NA
Hostility	7	552	94.9, <0.001	1.61 (1.35, 1.86)	0.13	1.49	0.51	0.32	9.08	<0.001	NA
Phobic anxiety	7	552	93.3, <0.001	1.42 (1.25, 1.59)	0.09	1.27	0.39	0.53	15.55	<0.001	NA
Paranoid ideation	7	552	90.7, <0.001	1.46 (1.31, 1.62)	0.08	1.44	0.47	0.07	2.10	0.044	NA
Psychoticism	7	552	93.9, <0.001	1.48 (1.30, 1.66)	0.09	1.33	0.39	0.53	15.22	<0.001	NA
*SARS survivors during the early stage of recovery*											
Somatization	4	291	0.0, 0.427	1.60 (1.51, 1.68)	0.04	1.42	0.44	0.57	17.00	<0.001	NA
Obsessive-compulsive	4	291	42.3, 0.158	1.76 (1.68, 1.85)	0.04	1.66	0.52	0.28	8.44	<0.001	NA
Interpersonal sensitivity	4	291	31.0, 0.226	1.55 (1.48, 1.63)	0.04	1.51	0.49	0.13	3.88	<0.001	NA
Depression	4	291	71.2, 0.015	1.65 (1.49, 1.81)	0.08	1.50	0.47	0.45	12.68	<0.001	NA
Anxiety	4	291	73.6, 0.010	1.57 (1.40, 1.74)	0.09	1.34	0.39	0.82	22.45	<0.001	NA
Hostility	4	291	59.5, 0.060	1.45 (1.34, 1.56)	0.06	1.49	0.51	0.11	3.20	0.002	NA
Phobic anxiety	4	291	41.7, 0.161	1.37 (1.29, 1.45)	0.04	1.27	0.39	0.37	10.96	<0.001	NA
Paranoid ideation	4	291	0.0, 0.404	1.37 (1.30, 1.44)	0.04	1.44	0.47	0.22	6.60	<0.001	NA
Psychoticism	4	291	71.7, 0.014	1.32 (1.20, 1.44)	0.06	1.33	0.39	0.02	0.64	0.325	NA
*SARS survivors during the late stage of recovery*											
Somatization	1	48	NA	1.65 (1.37, 1.93)	0.98	1.42	0.44	0.30	1.62	0.107	NA
Obsessive-compulsive	1	48	NA	1.59 (1.39, 1.79)	0.71	1.66	0.52	0.11	0.68	0.317	NA
Interpersonal sensitivity	1	48	NA	1.64 (1.43, 1.86)	0.76	1.51	0.49	0.20	1.18	0.199	NA
Depression	1	48	NA	1.55 (1.34, 1.76)	0.75	1.50	0.47	0.08	0.46	0.359	NA
Anxiety	1	48	NA	1.50 (1.30, 1.70)	0.72	1.34	0.39	0.28	1.53	0.123	NA
Hostility	1	48	NA	1.46 (1.18, 1.74)	0.98	1.49	0.51	0.04	0.21	0.390	NA
Phobic anxiety	1	48	NA	1.36 (1.11, 1.61)	0.89	1.27	0.39	0.13	0.70	0.312	NA
Paranoid ideation	1	48	NA	1.45 (1.21, 1.69)	0.84	1.44	0.47	0.01	0.08	0.398	NA
Psychoticism	1	48	NA	1.41 (1.14, 1.68)	0.95	1.33	0.39	0.11	0.58	0.337	NA

*NA* not applicable.

**Fig. 3:**
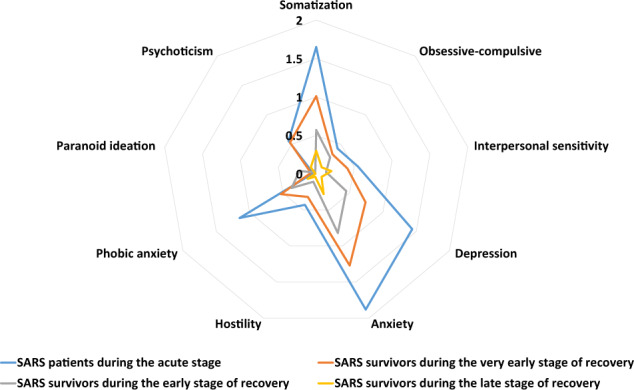
Severity of psychiatric symptoms of SARS patients and survivors. Radar map depicting the severity of psychiatric symptoms of SARS patients and survivors, as measured by Cohen’s d values.

With the exception of paranoid ideation (*d* = 0.04 and 0.07), the remaining eight psychiatric symptoms in acute SARS patients were medium-to-severe and symptom severity during very early recovery, while lower, had remained at medium-to-severe levels: somatization (*d* = 1.65 and 1.01), obsessive-compulsive (*d* = 0.43 and 0.33), interpersonal sensitivity (*d* = 0.55 and 0.41), depression (*d* = 1.44 and 0.74), anxiety (*d* = 1.88 and 1.27), hostility (*d* = 0.43 and 0.32), phobia (*d* = 1.15 and 0.53), and psychoticism (*d* = 0.56 and 0.53). Despite subsequent reductions in symptom severity in SARS, anxiety remained to be severe during early recovery (*d* = 0.82); somatization, obsessive-compulsive, depression, phobia, and paranoid ideation decreased to mild-to-medium during early recovery (*d* = 0.22–0.57); and somatization (*d* = 0.30) and anxiety (*d* = 0.28) remained at mild levels during late recovery (Table 2 and Fig. 3).

No significant publication bias was detected in meta-analyses of the SCL-90-R subscales of somatization, depression, anxiety, and hostility in acute patients with COVID-19 (*P* = 0.208–0.856; Table 2).

## Discussion

This study systematically summarized the profile of psychiatric symptoms in patients with COVID-19 and SARS, during both the acute and convalescent stages. We found that all ten SCL-90-R-defined psychiatric symptoms were exhibited in acute COVID-19 patients, and nearly all these symptoms were severe. Acute SARS patients, while they had less severe psychiatric symptoms than COVID-19 patients, still presented many medium-to-severe symptoms. Thereafter, there was a trend toward overall declines in severity of psychiatric symptoms observed in survivors of both COVID-19 and SARS. However, most psychiatric symptoms of COVID-19 (i.e., phobia, anxiety, and somatization) were still mild-to-medium during very early recovery, and some symptoms of SARS, such as somatization, interpersonal sensitivity, and anxiety, still remained mild during late recovery.

The underlying physiological and psychosocial mechanisms associated with human coronavirus diseases could explain the high psychiatric symptom burden in acute patients with COVID-19 and SARS. For example, researchers have found positive SARS-CoV-2 RNA and parainfectious/postinfectious inflammatory changes in the cerebrospinal fluid of COVID-19 patients^51,52^, so it is possible that psychiatric symptoms are part of the neuropsychiatric complications due to the central nervous system impact of viral infection^53^. Second, suffering from coronavirus diseases per se, a potentially life-threatening illness, is a stressful event for patients. Due to this, fear of death, worry about the infection of family members, despair, anger, frustration, and insomnia are common stress reactions in this patient population^5^. Third, physical discomfort and pain caused by COVID-19 and SARS could further exacerbate emotional reactions to coronavirus diseases. Fourth, because of the isolation treatment for patients, separation from family members and friends would increase the risk of feeling lonely and other mental health problems^54^. Fifth, antiviral treatment may also contribute to the psychiatric manifestations of patients; for example, there is evidence that both chloroquine and steroids could induce psychotic episodes^55,56^. We speculate that various severe psychiatric symptoms of COVID-19 are more likely to be the result of the joint effects of the abovementioned factors.

The relatively lower severity of psychiatric symptoms in SARS than in COVID-19 patients might be ascribed to the different psychosocial impacts of the two coronavirus diseases. For example, the SARS epidemic mainly affected people of Asian countries, but the COVID-19 pandemic has been a global crisis, affecting people of nearly all countries in the world. Compared to SARS, COVID-19 has quicker and wider transmission, disproportionate effects on older adults, and a high case-fatality rate in older adults^57^, and therefore, COVID-19 patients may have higher levels of psychological distress and fears of death than SARS patients. This may also explain why phobia was the most severe psychiatric symptom in COVID-19 patients. Because of physical complications and discomfort caused by coronavirus diseases, severe symptoms of somatization with acute COVID-19 and SARS are expected. In addition, unlike the SARS epidemic in 2003, the ongoing COVID-19 pandemic is occurring concurrently with an “infodemic”, where misinformation and disinformation can be easily and quickly disseminated via social media platforms^58^, which may further exacerbate the poor mental health of COVID-19 patients.

The reduced severity of psychiatric symptoms in COVID-19 and SARS patients from the acute stage to the late recovery stage suggests that psychiatric symptoms in the acute stage are mainly acute stress reactions and are therefore transient. These results indicate the importance of early mental health and psychosocial services at the stage of inpatient treatment. Nevertheless, the persistence of several psychiatric symptoms in SARS survivors throughout recovery might suggest the necessity of additional psychiatric symptom assessment and mental health services for the rehabilitation of COVID-19 survivors. Some postdischarge psychosocial factors may increase the risk of depression and other mental health problems in SARS survivors; for example, stigma associated with SARS and financial loss or even unemployment due to the past history of SARS infection. A recently published prognosis study reported that COVID-19 survivors were still suffering from fatigue, muscle weakness, sleep difficulties, depression, and anxiety 6 months after acute infection^16^. These findings are consistent with the residual psychiatric symptoms in recovered COVID-19 and SARS survivors in our study, such as somatization and anxiety.

This study has some limitations. First, the quality assessment results of the included studies suggest that, to a certain extent, these included studies are at risk of bias, so we must be cautious in generalizing the study findings. Second, owing to the lack of SCL-90-R data from COVID-19 survivors during late recovery, and the very limited SCL-90-R data from SARS survivors during late recovery, more studies are warranted to investigate psychiatric symptoms of COVID-19 survivors, particularly long-term prospective studies. Third, since most of the included studies excluded severe or critical patients with coronavirus diseases, our study may underestimate the severity of psychiatric symptoms of patients with COVID-19 and SARS. Fourth, the present study focused on psychiatric symptoms based only on a self-rating scale only, the SCL-90-R, so data on psychiatric symptoms of COVID-19 patients who were illiterate and cognitively impaired were unavailable. Future studies using detailed comprehensive psychiatric interviews would provide a more comprehensive picture of the profile of psychiatric symptoms of COVID-19. Fifth, the participants in the studies included in this meta-analysis were all Chinese patients with coronavirus diseases. Because sociocultural factors play an important role in the clinical manifestations of mental health problems, caution is needed when generalizing our results to COVID-19 patients in countries other than China. Finally, as shown in Table 2, high levels of heterogeneity were detected in most meta-analyses. However, due to the limited number of included studies in each meta-analysis, we were not able to perform subgroup analysis to identify factors associated with the severity of each psychiatric symptom. It is worth noting that our study provided only an overall profile of the psychiatric symptoms of COVID-19, not a detailed profile of psychiatric symptoms.

In summary, a wide spectrum of severe psychiatric symptoms occur in COVID-19 patients, and most symptoms are still mild-to-medium during very early recovery. Based on SCL-90-R data from SARS patients and survivors, the severity of psychiatric symptoms of COVID-19 may decline following discharge, but some symptoms could persist for a long time during the convalescent stage. These findings suggest the urgent need of patients for extensive mental health services and psychological crisis intervention during the acute stage of COVID-19. Furthermore, it is also important to periodically monitor the psychiatric symptoms and provide psychosocial support, and psychiatric consultation and treatment (when necessary) for COVID-19 survivors during their convalescent stage. In addition, more research is needed to examine the longitudinal changes in psychiatric symptoms of COVID-19 survivors.
